# Motor dysfunction in cerebellar Purkinje cell-specific vesicular GABA transporter knockout mice

**DOI:** 10.3389/fncel.2013.00286

**Published:** 2014-01-16

**Authors:** Mikiko Kayakabe, Toshikazu Kakizaki, Ryosuke Kaneko, Atsushi Sasaki, Yoichi Nakazato, Koji Shibasaki, Yasuki Ishizaki, Hiromitsu Saito, Noboru Suzuki, Nobuhiko Furuya, Yuchio Yanagawa

**Affiliations:** ^1^Department of Genetic and Behavioral Neuroscience, Gunma University Graduate School of MedicineMaebashi, Japan; ^2^Japan Science and Technology AgencyCREST, Tokyo, Japan; ^3^Department of Otolaryngology-Head and Neck Surgery, Gunma University Graduate School of MedicineMaebashi, Japan; ^4^Institute of Experimental Animal Research, Gunma University Graduate School of MedicineMaebashi, Japan; ^5^Department of Pathology, Saitama Medical UniversityMoroyama, Japan; ^6^Department of Human Pathology, Gunma University Graduate School of MedicineMaebashi, Japan; ^7^Department of Molecular and Cellular Neurobiology, Gunma University Graduate School of MedicineMaebashi, Japan; ^8^Department of Animal Genomics, Functional Genomics Institute, Mie University Life Science Research CenterTsu, Japan

**Keywords:** cerebellum, Purkinje cells, VGAT, knockout mice, ataxia, mouse model

## Abstract

γ-Aminobutyric acid (GABA) is a major inhibitory neurotransmitter in the adult mammalian central nervous system and plays modulatory roles in neural development. The vesicular GABA transporter (VGAT) is an essential molecule for GABAergic neurotransmission due to its role in vesicular GABA release. Cerebellar Purkinje cells (PCs) are GABAergic projection neurons that are indispensable for cerebellar function. To elucidate the significance of VGAT in cerebellar PCs, we generated and characterized PC-specific VGAT knockout (L7-VGAT) mice. *VGAT* mRNAs and proteins were specifically absent in the 40-week-old L7-VGAT PCs. The morphological characteristics, such as lamination and foliation of the cerebellar cortex, of the L7-VGAT mice were similar to those of the control littermate mice. Moreover, the protein expression levels and patterns of pre- (calbindin and parvalbumin) and postsynaptic (GABA-A receptor α1 subunit and gephyrin) molecules between the L7-VGAT and control mice were similar in the deep cerebellar nuclei that receive PC projections. However, the L7-VGAT mice performed poorly in the accelerating rotarod test and displayed ataxic gait in the footprint test. The L7-VGAT mice also exhibited severer ataxia as VGAT deficits progressed. These results suggest that VGAT in cerebellar PCs is not essential for the rough maintenance of cerebellar structure, but does play an important role in motor coordination. The L7-VGAT mice are a novel model of ataxia without PC degeneration, and would also be useful for studying the role of PCs in cognition and emotion.

## INTRODUCTION

γ-Aminobutyric acid (GABA) is a major inhibitory neurotransmitter and is a trophic factor in the central nervous system (CNS) (Owens and Kriegstein, 2002; Represa and Ben-Ari, 2005). The activities of GABA are mediated by vesicular or non-vesicular release after GABA is synthesized by glutamate decarboxylase (GAD) (Semyanov et al., 2004; Buddhala et al., 2009). Vesicular GABA release is accomplished after GABA is transported into the synaptic vesicles by the vesicular GABA transporter (VGAT) and subsequently exocytosed (McIntire et al., 1997; Sagné, 1997). Thus, VGAT is crucial for the vesicular GABA release, and VGAT deficits cause the nearly complete disappearance of inhibitory postsynaptic currents (IPSCs) in dissociated cultures or slices derived from VGAT knockout (KO) mice (Wojcik et al., 2006; Saito et al., 2010). However, because VGAT KO mice die during the perinatal period, the study of VGAT function in the adult CNS has been limited (Tong et al., 2008).

The cerebellum is important for motor function, cognition, and emotion (Strata et al., 2011; Lisberger and Thach, 2013). Cerebellar Purkinje cells (PCs) are the sole output neurons of the cerebellar cortex and project to the deep cerebellar nuclei (DCN) and vestibular nuclei (VN) neurons. The DCN and VN are involved in motor control in animals through their communication with the nuclei of the thalamus and brainstem. Because PCs are GABAergic neurons, it is thought that the existence of VGAT in PCs is critical for PC function. However, the significance of VGAT in PCs has not been demonstrated.

Here, to study the roles of VGAT in PCs, we generated cerebellar PC-specific VGAT KO (L7-VGAT) mice and examined the cerebellar structure and motor function of these mice, as indices of the developmental failure and dysfunction of adult PCs, respectively. In these knockout mice, the overall cerebellar structure was not altered, but motor discoordination was observed. These results indicate that VGAT in cerebellar PCs is critical for motor coordination in animals and the L7-VGAT mice are a novel mouse model of cerebellar dysfunction without apparent structural alterations in the cerebellum.

## MATERIALS AND METHODS

### MICE

We generated heterozygous mice carrying one floxneo allele (*VGAT*^floxneo/^^+^ mice) in which the 5′-loxP site was introduced into the *Xba*I site in intron 1 and an frt-flanked PGK-neo cassette followed by the 3′-loxP sequence was inserted into the *Kpn*I site in the 3′-flanking region (Ebihara et al., 2003; Saito et al., 2010). The *VGAT*^floxneo/^^+^ mice were mated to FLP66 transgenic mice (Takeuchi et al., 2005), and the male offspring were further crossed with C57BL/6 mice to eliminate the PGK-neo gene from the genome through Flp/frt-mediated excision. The resultant *VGAT*^flox/^^+^ mice were intercrossed to generate *VGAT*^flox/flox^ mice in which exons 2 and 3 of the *VGAT* gene were flanked by loxP sites. The generation of L7-Cre knock-in mice has been previously described (Saito et al., 2005). In these mice, the Cre recombinase gene followed by a PGK-neo cassette was inserted into exon 2 of the *L7/Pcp-2 (L7)* gene, and the Cre recombinase is expressed under the control of the endogenous *L7* promoter. *VGAT*^flox/flox^ mice were crossed with heterozygous L7-Cre knock-in mice (*L7*^Cre/^^+^ mice) to obtain *VGAT*^flox/flox^; *L7*^Cre/^^+^ mice. *VGAT*^flox/flox^; *L7*^Cre/^^+^ mice were mated to *VGAT*^flox/flox^; *L7*^+^^/^^+^ mice to obtain *VGAT*^flox/flox^; *L7*^Cre/^^+^ mice and *VGAT*^flox/flox^; *L7*^+^^/^^+^ mice, which were used in the subsequent experiments. We refer to the *VGAT*^flox/flox^; *L7*^Cre/^^+^ mice and the *VGAT*^flox/flox^; *L7*^+^^/^^+^ mice as L7-VGAT mice and control mice, respectively, hereafter.

All animal procedures were conducted in accordance with the guidelines of the NIH and were reviewed and approved by the Animal Care and Experimentation Committee of Gunma University, Showa Campus (Maebashi, Japan). Every effort was made to minimize the number of animals used and their suffering.

### GENOTYPING BY PCR

PCR genotyping was performed using tail genomic DNA. Genotypes of the *VGAT*^+^^/^^+^, *VGAT*^flox/^^+^, and *VGAT*^flox/flox^ mice were determined by PCR using the following oligonucleotides: primer 1 (5′-TCAGAGGCTTCTTCCTAGGGCTGCTG-3′) and primer 2 (5′-GACCTCCCCCATTGCATAGAATGGCAC-3′), which correspond to the 3′-flanking region of the VGAT gene. Primers 1 and 2 amplified a 183-bp fragment specific for the wild-type allele and a 310-bp fragment specific for the floxed allele. The following primers were used for the *L7*^+^^/^^+^, *L7*^Cre/^^+^, and *L7*^Cre/Cre^ genotype mice: primer 3 (5′-CCAGGAAGGCTTCTTCAACCTGCT-3′), primer 4 (5′-CCTGGGTGTTGACCAGCATATCC-3′), and primer 5 (5′-GTACGGTCAGTAAATTGGACATGCGG-3′). Primers 3 and 4 amplified a 311-bp fragment specific for the wild-type allele, and primers 3 and 5 yielded a 152-bp fragment specific for the Cre knock-in allele (Saito et al., 2005).

### ANATOMICAL ANALYSIS WITH CRESYL VIOLET STAINING

The whole brains of mice at postnatal 40 weeks of age (P40W) were removed and embedded in Tissue-Tek OCT compound (Sakura Finetek Japan, Tokyo, Japan). Samples were sectioned on a cryostat (CM3050S, Leica, Nussloch, Germany) at 10 μm. The cryosections were mounted onto SuperFrost Plus microscope slides (Matsunami Glass, Osaka, Japan), air-dried for more than 20 min at room temperature, and stored at –80°C until use. The thicknesses of the granule cell and molecular layers and the densities of the PC somata were determined using sections stained with cresyl violet, and two sections were prepared from each animal. Laminar thickness was measured at five points along the rostral aspect of lobules IV–V in each section. The densities of the PC somata were determined by dividing the total number of PCs per section in lobule III by the length of the entire PC layer in this lobule. PCs were defined as cells with diameters greater than 15 μm. The data obtained from four control and four L7-VGAT mice were averaged.

### DOUBLE IN SITU HYBRIDIZATION FOR VGAT AND CALBINDIN

To detect murine *VGAT* transcripts (GenBank accession number NM_009508, nucleotides 878–2499), *VGAT* cRNA probes were synthesized using a digoxigenin (DIG) RNA Labeling Kit (Roche Diagnostics, Basel, Switzerland) and T3 RNA polymerase (Roche Diagnostics). To detect murine *calbindin* (*CB*) transcripts (GenBank accession number NM_009788, nucleotides 110–994), *CB* cRNA probes were synthesized using a FITC-dUTP labeling kit (Roche Diagnostics) and Sp6 RNA polymerase (Roche Diagnostics) according to the manufacturer’s instructions.

The whole brains of mice at P2W, P4W, P8W, P16W, and P40W were removed and embedded in Tissue-Tek OCT compound. Samples were sectioned on a cryostat at 10 μm. The cryosections were mounted onto SuperFrost Plus microscope slides, air-dried for more than 20 min at room temperature, and stored at -80°C until use. The cryosections were fixed with 4% paraformaldehyde (PFA) for 10 min, and serial sections were treated according to previously described protocols (Watakabe et al., 2007, 2010). The *VGAT* and *CB* transcripts signals were obtained using a fluorescent microscope (BZ-8000, Keyence, Osaka, Japan).

### EVALUATION OF VGAT KNOCKOUT IN PCS

The presence of VGAT in the L7-VGAT PCs was determined by the detection of *in situ* hybridization (ISH) signals for both *VGAT* and *CB*. The percentage of *VGAT* mRNA-positive PCs was determined in lobule III, because lobule III was uniformly stained with our ISH protocol. We analyzed two sections per animal and used two animals per genotype at 2, 4, 8, 16, and 40 weeks of age (postnatal). The number of *VGAT* mRNA-positive PCs was divided by the total number of PCs. Because CB is a marker of PCs (Celio, 1990), *CB*-positive cells were regarded as PCs.

### IMMUNOHISTOCHEMISTRY

The primary antibodies used in this study included rabbit anti-VGAT (1/50; Takayama and Inoue, 2004a), mouse anti-CB (1/1,000; Swant, Bellinzona, Switzerland), mouse anti-parvalbumin (PV; 1/5,000; Sigma-Aldrich, Saint Louis, MO, USA), guinea pig anti-GABA_A_ receptor α1 subunit (GABA_A_Rα1; 1/2,500; Takayama and Inoue, 2004b), and mouse anti-gephyrin (1/1,000; Synaptic Systems, Goettingen, Germany). Secondary antibodies included Alexa Fluor 488 anti-rabbit IgG (H + L; 1/200; Molecular Probes/Invitrogen, Eugene, OR, USA), Alexa Fluor 568 goat anti-mouse IgG (H + L; 1/200; Molecular Probes/Invitrogen), and Alexa Fluor 488 goat anti-guinea pig IgG (H + L; 1/1,000; Molecular Probes/Invitrogen).

To detect VGAT, CB, and PV, mice at P40W were transcardially perfused with 4% PFA in 0.1 M phosphate buffer, pH 7.4 (PB), under deep anesthesia with diethyl ether. Brains were harvested from the perfused animals, post-fixed in the identical fixative for 12 h at 4°C, and immersed in 30% sucrose in 0.1 M PB and 0.05% NaN_3_ for at least 48 h for cryoprotection. The frozen brains were sliced at 20 μm using a cryostat, then mounted onto slides, and dried for more than 30 min at room temperature. Immunostaining for VGAT and CB was performed following a previously published protocol (Takayama and Inoue, 2004a). Alexa568- or Alexa488-conjugated secondary antibodies were used to detect the primary antibodies. To detect PV, the sections were blocked with 3% bovine serum albumin (BSA) in 0.3% Triton X-100 in PBS (PBSTx) for 1 h and then incubated with an anti-PV antibody at 4°C overnight. The primary antibody was detected with an Alexa Fluor 568 anti-rabbit IgG. To detect the colocalization of GABA_A_Rα1 and gephyrin, double immunofluorescence staining was performed in sections prepared from fresh-frozen tissue as previously described for the ISH. Sections were fixed in PB containing 0.5% PFA for 10 min, blocked with 3% normal goat serum in PB for 1 h, and then incubated overnight at 4°C with anti-GABA_A_Rα1 and anti-gephyrin antibodies. The primary antibodies were detected using an anti-guinea pig Alexa Fluor 488 and anti-mouse Alexa Fluor 568-conjugated secondary antibodies. Fluorescence signals were obtained using an optical microscope (AxioCam, Carl Zeiss, Jena, Germany). The composite images were prepared from the digital data files using Adobe Photoshop and Illustrator.

### WESTERN BLOT ANALYSIS

Whole cerebella of 40-week-old control and L7-VGAT mice were coronally cut at the most rostral level of the DCNs, and both sides of the DCNs were separated from the sectioned cerebella by cutting between the DCNs and the cerebellar cortex in ice-cold PBS. Both sides of the DCN regions and the residual tissues from one animal were defined as DCN and cerebellar cortex samples, respectively. The DCN and cerebellar cortex samples were homogenized in ice-cold homogenization buffer (320 mM sucrose, 50 mM Tris–HCl, pH 7.2, 5 mM ethylenediaminetetraacetic acid (EDTA), and 1 mM phenylmethylsulfonyl fluoride (PMSF)). Homogenates were centrifuged at 3,000 rpm for 10 min at 4°C to obtain the supernatant as a crude fraction. The protein concentrations of the crude fractions were determined using the BCA protein assay reagent (Pierce, Rockford, IL, USA) and BSA as a standard. Equal amounts of protein from the crude fractions of the DCNs or cerebellar cortices were separated using 10% sodium dodecyl sulfate–polyacrylamide gel electrophoresis (SDS–PAGE) to detect VGAT, calbindin, gephyrin, and GABA_A_Rα1 and 13% SDS–PAGE to detect GAT3, β-actin, and parvalbumin. Subsequently, the proteins were transferred to nitrocellulose membranes using a semidry transfer method. The membranes were reacted with rabbit anti-VGAT (1/100; Takayama and Inoue, 2004a), mouse anti-calbindin (1/100; Swant), rabbit anti-gephyrin (1/1,000; Synaptic Systems, Goettingen, Germany), guinea pig anti-GABA_A_Rα1 (1/100; Takayama and Inoue, 2004b), rabbit anti-GAT3 (1/100; Chemicon/Millipore, Tokyo, Japan), mouse anti-β-actin (1/10,000; Abcam, Tokyo, Japan), and rabbit anti-parvalbumin (1/30,000; Swant, Switzerland). The secondary antibodies included horseradish peroxidase-conjugated goat anti-rabbit, mouse or guinea pig IgG (1/10,000; Jackson Immunoresearch, West Grove, PA, USA). Signals were visualized by using an Enhanced Chemiluminescence (ECL) Western Blotting Analysis System (GE Healthcare Life Sciences, Buckinghamshire, UK) followed by light-capture imaging (ATTO, Tokyo, Japan). The images were scanned using the NIH IMAGE software to quantify the protein levels.

### ACCELERATING ROTATING ROD TEST

The rotating rod apparatus (Accelerating Model, Ugo Basile, Biological Research Apparatus, Varese, Italy) was used to evaluate the motor skill abilities of the mice. Male mice at postnatal 8, 16, and 40 weeks were tested with three trials per day for three consecutive days. Each trial lasted a maximum of 5 min during which time the rotating rod linearly underwent accelerated from 4 to 40 rpm. Animals were scored for their latency to fall (in seconds) in each trial. The animals were allowed at least 30 min of rest between trials to avoid fatigue and exhaustion. Statistical analyses were performed using two-way or one-way repeated measures ANOVA with the factors of genotype and trial.

### FOOTPRINT TEST

The footprint test was performed to examine the gaits of the mice. The rear paws of the mice were inked, and the mice were allowed to walk along a 50-cm-long, 10-cm-wide runway (with 10-cm-high walls). A fresh sheet of paper was placed on the floor of the runway for each run. Each mouse was tested twice at P16W and P40W. The footprint patterns of the hind paws were evaluated in terms of the following two parameters (both measured in centimeters): (1) stride length, which was taken as the average distance between each consecutive pair of footprints for each hind paw; and (2) stride width, which was taken as the average perpendicular distance between each footprint and the line connecting each pair of consecutive footprints on the opposite side. Statistical analysis was performed using Student’s *t*-test.

## RESULTS

### GENERATION OF L7-VGAT MICE

**FIGURE 1:**
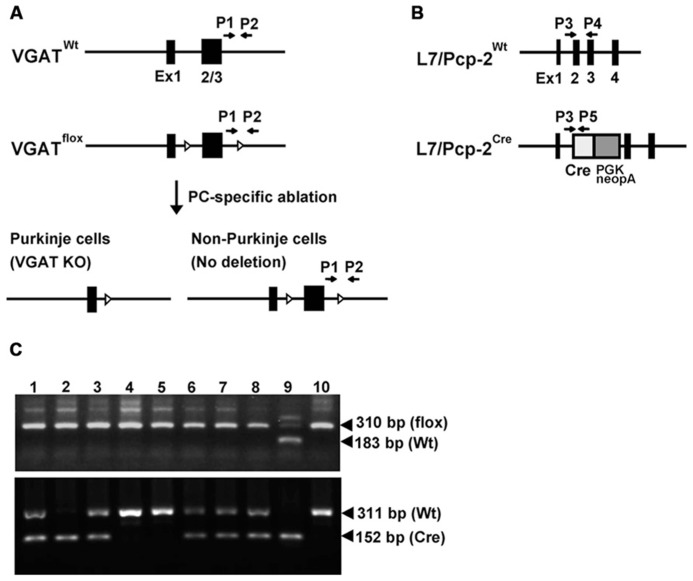
***L7/Pcp-2* promoter-mediated disruption of the VGAT gene.** **(A)** Schema of the wild-type and floxed *VGAT* allele (upper). Black boxes and white triangles indicate exons and loxP sites in the *VGAT* gene, respectively. Black arrows indicate the primer sites for PCR genotyping. Schema of cerebellar Purkinje cell (PC)-specific *VGAT* gene disruption in L7-VGAT mice (lower). **(B)** Schema of the wild-type and Cre knock-in *L7/Pcp-2* (*L7*) allele. Black boxes indicate exons in the *L7* gene. White and gray boxes indicate the Cre gene and the PGK-Neo cassette, respectively, that were introduced into exon 2 of the *L7* gene. The black arrows indicate the primer sites for PCR genotyping. **(C)** PCR genotyping of littermates (numbers 1–8) obtained by crossing a *VGAT*^flox/flox^; *L7*^Cre/^^+^ mouse with a *VGAT*^flox/flox^; *L7*^+^^/^^+^ mouse. Upper and lower panels show the genotyping of the *VGAT* and *L7* gene, respectively. Lanes 1–8 in both panels correspond to the identical littermate number. Lanes 9 and 10 in both panels were used as controls. Lanes 9 and 10 in the upper panel indicate the *VGAT*^flox/^^+^ and *VGAT*^flox/flox^ genotypes, respectively. Lanes 9 and 10 in the lower panel indicate the *L7*^Cre/Cre^ and *L7*^+^^/^^+^ genotypes, respectively.

To investigate the role of VGAT in cerebellar PCs on cerebellar structure and motor function, we generated cerebellar PC-specific VGAT KO mice. We used floxed VGAT mice in which exons 2 and 3 of the *VGAT* gene are flanked by loxP sequences (Saito et al., 2010) and L7-Cre knock-in mice that express the Cre recombinase gene under the control of the intrinsic *L7* promoter (Saito et al., 2005) (**Figures 1A,B**). We crossed *VGAT*^flox/flox^; *L7*^+^^/^^+^ mice with *VGAT*^flox/flox^; *L7*^Cre/^^+^ mice to obtain *VGAT*^flox/flox^; *L7*^+^^/^^+^ and *VGAT*^flox/flox^; *L7*^Cre/^^+^ mice (**Figure 1C**). We refer to *VGAT*^flox/flox^; *L7*^+^^/^^+^ and *VGAT*^flox/flox^; *L7*^Cre/^^+^ as the control and L7-VGAT mice, respectively, hereafter. In the L7-Cre knock-in mice, Cre recombinase is specifically expressed in the cerebellar PCs and retinal cells including the bipolar neurons and photoreceptor cells (Saito et al., 2005). On the other hand, VGAT is expressed in PCs, but not in these retinal cells. Thus, in the L7-VGAT mice, VGAT was expected to be disrupted only in the PCs. The L7-VGAT mice were born and grew until 16 postnatal weeks (P16W) without any apparent abnormal phenotype. At P40W, the L7-VGAT mice were fertile and normal in terms of nursing, but they displayed abnormal ambulation. These mice walked awkwardly and easily fell off the edge of a table. The L7-VGAT mice survived until they were more than 1 year of age.

### PC-SPECIFIC AND AGE-DEPENDENT DISAPPEARANCE OF VGAT mRNA IN L7-VGAT MICE

**FIGURE 2:**
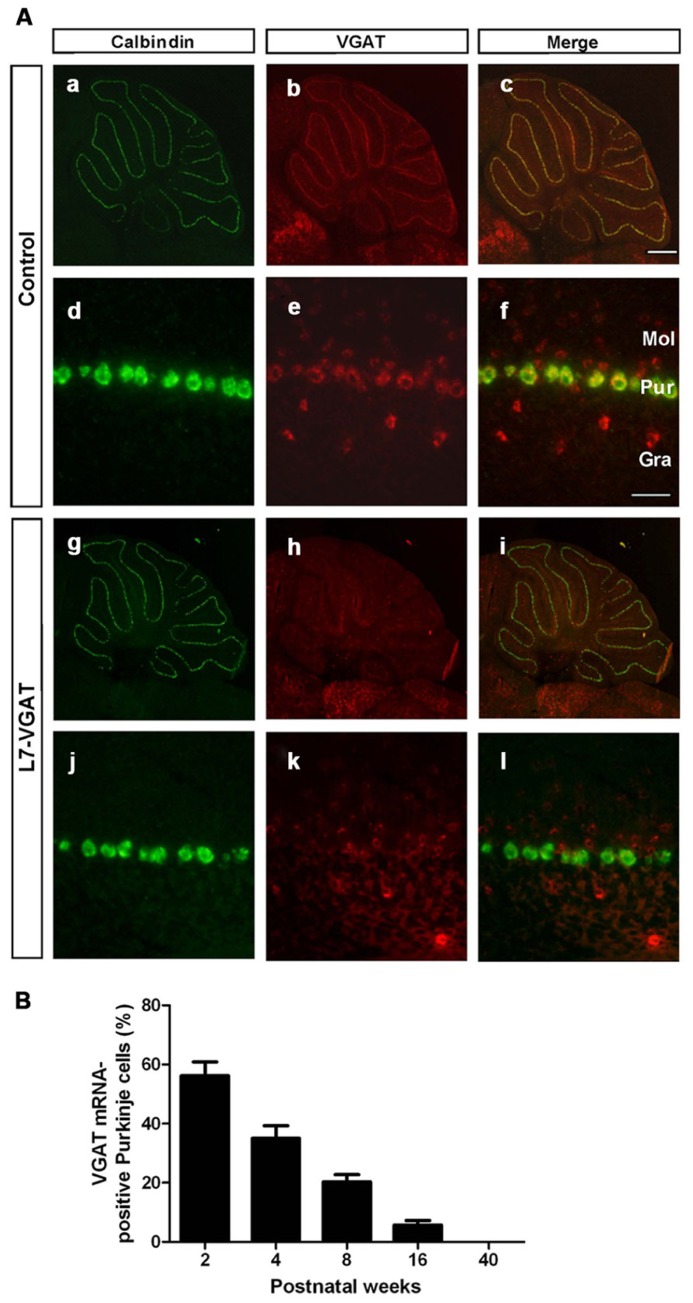
**Disappearance of *VGAT* mRNA specifically in the Purkinje cells of L7-VGAT mice. (A)** Double *in situ* hybridizations for *calbindin D-28K* (a,d,g,j; green) and *VGAT* (b,e,h,k; red) were performed in parasagittal sections of 40-week-old mice. The cerebella of control (a–f) and L7-VGAT (g–l) mice are shown. (a–c) and (g–i) show images of the whole cerebella. (d–f) and (j–l) show images of lobule III. Merged images of (a,d,g,j) and (b,e,h,k) are shown in (c,f,i,l). Bars represent 500 μm (c) and 50 μm (f). **(B)** The percentage of *VGAT* mRNA-positive Purkinje cells (PCs) in the L7-VGAT mice at 2, 4, 8, 16, and 40 weeks postnatal. The number of *VGAT* mRNA-positive Purkinje cells was divided by the total number of *calbindin* mRNA-positive PCs in lobule III (L7-VGAT, *n* = 2; control, *n* = 2 of each age). The total numbers of calbindin-positive cells in the L7-VGAT mice that were counted were 230 at 2 weeks postnatal (P2W), 264 at P4W, 243 at P8W, 232 at P16W, and 200 at P40W. Each value represents the mean± SEM. Gra, granule cell layer; Pur, Purkinje cell layer; and Mol, molecular layer.

To assess whether *VGAT* mRNA was specifically lost in the PCs of the L7-VGAT mice, we performed double ISH for *VGAT* and *calbindin* (*CB*) (**Figure 2**). CB was used as a marker of PCs because CB is specifically expressed in PCs in the cerebellar cortex and DCNs (Celio, 1990). In the control cerebellum, hybridization signals for *VGAT* mRNA were lined up along the PC layer and completely overlapped with the CB signals (**Figures 2Aa–f**). In contrast, no cells containing *CB* mRNA expressed *VGAT* mRNA in the L7-VGAT cerebellum at P40W (**Figures 2Ag–l**). However, other *VGAT* mRNA signals were found in the molecular and granule cell layers of the P40W L7-VGAT mice. Furthermore, *VGAT* mRNA signals in brain regions outside of the cerebellum did not appear to be different between coronal sections of L7-VGAT and control mice at either P16W or P40W (data not shown).

Next, to assess the time at which *VGAT* mRNA was lost in the L7-VGAT PCs during development, we performed double ISH for *VGAT* and *CB* and counted the number of *VGAT*-positive PCs relative to the total number of PCs at five postnatal time points (i.e., P2W, P4W, P8W, P16W, and P40W; **Figure 2B**). The percentage of *VGAT*-positive PCs relative to the total PCs gradually decreased with age from 56% at P2W to 0% at P40W (**Figure 2B**).

### LOSS OF VGAT PROTEIN IN L7-VGAT PCS

**FIGURE 3:**
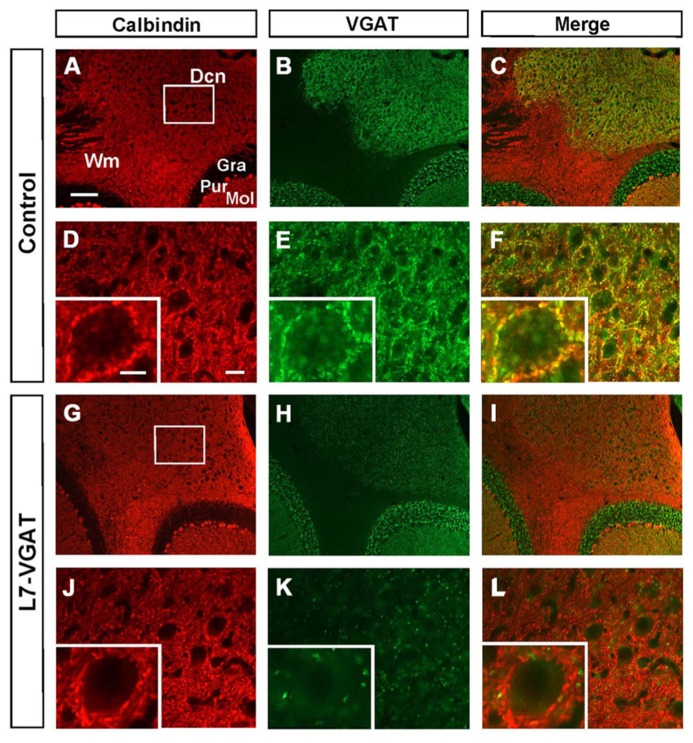
**Loss of calbindin- and VGAT-double immunoreactive signals in the L7-VGAT DCN.** Double immunohistochemical staining for calbindin **(A,D,G,J**; red) and VGAT **(B,E,H,K**; green) was performed for coronal sections of the cerebella of 40-week-old control **(A–F)** and L7-VGAT **(G–L)** mice. Areas surrounded by white rectangles in **(A)** and **(G)** are shown in **(D)** and **(J)**, respectively. Insets in **(D–F, J–L)** illustrate typical somata for each area. **(E,F)** and **(K,L)** represent areas that are identical to those in **(D)** and **(J)**, respectively. Merged images of **(A,D,G,J)** and **(B,E,H,K)** are shown in **(C,F,I,L)**. Scale bars represent 100 μm **(A)**, 20 μm **(D)** and 10 μm (inset of **(D)**). Dcn, deep cerebellar nucleus; Gra, granule cell layer; Pur, Purkinje cell layer; Mol, molecular layer; and Wm, white matter.

To confirm whether VGAT protein was also lost, we performed immunohistochemistry for VGAT and CB in the L7-VGAT PCs (**Figure 3**). CB is a PC marker protein that is located in the soma, dendrites, axonal fibers, and axon terminals of PCs (Bäurle et al., 1997). CB-immunoreactive signals were present in the white matter, the DCNs, and the cerebellar cortices of both control and L7-VGAT mice (**Figures 3A,D,G,J**). VGAT-immunoreactive signals overlapped with CB signals in the DCNs of the control mice, which suggests that the VGAT signals were derived from PCs. The DCN neurons appeared to be heavily innervated by GABAergic terminals of the PCs (**Figure 3F**). In the L7-VGAT DCNs at P40W, the VGAT protein signals that were positive for CB had nearly disappeared (**Figure 3L**). In contrast, the VGAT signals in the cerebellar cortices of both genotypes were similar (**Figures 3B,H**). These results demonstrate that our L7-VGAT mice exhibited cerebellar PC-specific VGAT knockout at P40W. In the L7-VGAT DCNs at P40W, a small number of VGAT-positive and CB-negative signals remained. These results are consistent with a previous report that a large portion of inhibitory synapses on DCN neurons is derived from PCs (Garin et al., 2002).

**FIGURE 4:**
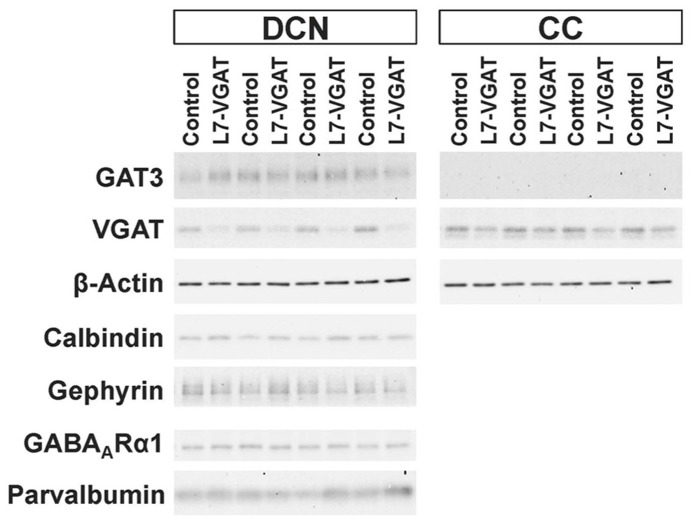
**Significant reduction in VGAT protein and unaltered levels of other pre- and postsynaptic proteins in the L7-VGAT DCN.** Western blot analyses of GAT3, β-actin, calbindin, gephyrin, GABA_A_Rα1, and parvalbumin proteins in the crude fractions from control and L7-VGAT DCN and cerebellar cortices at P40W. DCN, deep cerebellar nuclei; and CC, cerebellar cortex.

To verify our immunohistochemical results, we performed Western blot analyses (**Figure 4**; **Table 1**). To acquire DCN tissue for Western blot analyses, we focused on GAT3. GAT3 is one of the GABA transporters, which uptake extracellular GABA, and is located in the glial cell membrane (Itouji et al., 1996). It has been reported that the DCNs exhibit intense GAT3 immunoreactivity and that this immunoreactivity is faint in the cerebellar cortex (Itouji et al., 1996). Therefore, we used GAT3 as a marker of the DCN region. We dissected the cerebellum into the putative DCN and cerebellar cortex regions (see section “Materials and Methods”). Although GAT3 signals were detected in the crude fractions of control and L7-VGAT DCNs, they were not detected in the fractions of the control or L7-VGAT cerebellar cortices. On the basis of the results, we judged that our method enabled to separate the DCN from the cerebellar cortex. VGAT protein levels were drastically reduced in the DCNs of the L7-VGAT mice, and this result was consistent with that of the immunohistochemical study shown in **Figure 3**. In addition, VGAT signal in the L7-VGAT cerebellar cortex was also significantly reduced. It was expected that the reduction of the VGAT signal in the crude fraction of the L7-VGAT cerebellar cortex would reflect the loss of VGAT protein in the PCs of the cerebellar cortical sample due to the loss of the transport of VGAT protein to the axon terminals.

**Table 1:** Relative signal intensities of protein immunoreactiveties.

	DCN		CC	
	Control	L7-VGAT	Control	L7-VGAT
VGAT	100± 18	40± 6**	100± 10	70± 14*
Calbindin	100± 10	121± 19		
Gephyrin	100± 13	96± 29		
GABA_A_Rα1	100± 15	107± 12		
Parvalbumin	100± 15	114± 18		

Signal intensities of each protein were normalized by those of β-actin. Values represent mean± SD (*n* = 4). **p < 0.001, *p < 0.05 (Student’s *t*-test).

### ROUGHLY MAINTAINED-STRUCTURE OF THE L7-VGAT CEREBELLUM

**FIGURE 5:**
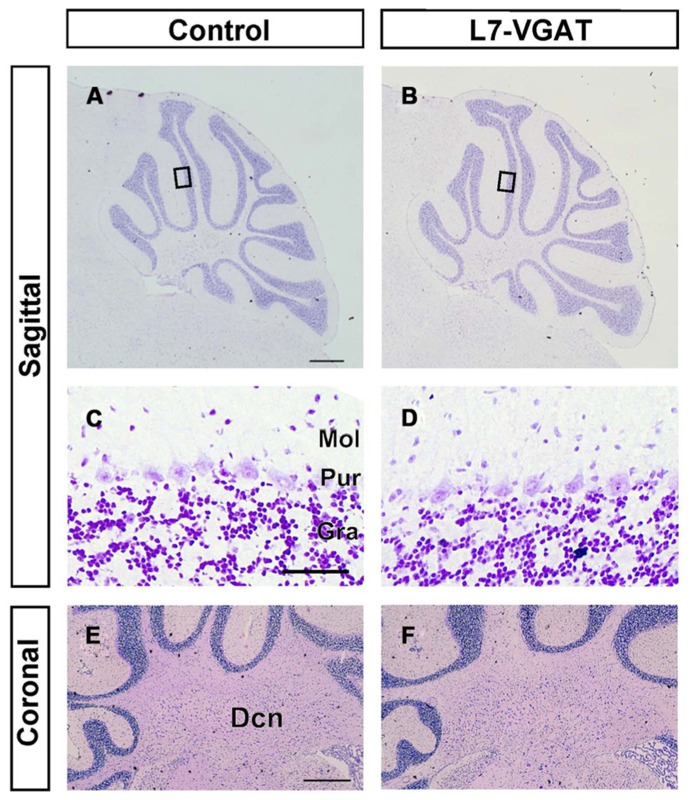
**Roughly maintained-structure of the L7-VGAT cerebellum.** Cresyl violet staining of sagittal **(A–D)** and coronal **(E,F)** sections of the whole cerebellum **(A,B)**, cerebellar cortex **(C,D)**, and DCN **(E,F)** of 40-week-old control **(A,C,E)** and L7-VGAT **(B,D,F)** mice. Areas surrounded by black rectangles in **(A)** and **(B)** are shown in **(C)** and **(D)**, respectively. Scale bars represent 500 μm **(A,E)** and 50 μm **(C)**. Dcn, deep cerebellar nucleus; Gra, granule cell layer; Pur, Purkinje cell layer; and Mol, molecular layer.

It is known that GABA can be involved in neural development (Owens and Kriegstein, 2002; Represa and Ben-Ari, 2005). Thus, it is possible that the anatomical structure of the L7-VGAT cerebellum was altered. To examine whether the L7-VGAT cerebellum was anatomically affected, we performed cresyl violet staining (**Figure 5**). We did not detect any overt abnormalities in the lamination or foliation of the 40-week-old L7-VGAT cerebellum (**Figures 5A,B**). The sizes, densities, and staining intensities of the cells in each layer of the L7-VGAT cerebellar cortex were similar to those in control cerebellum (**Figures 5C,D**). The overall size of DCN appeared to remain unaltered (**Figures 5E,F**). Additionally, there were no significant differences in the thicknesses of the molecular or granule cell layers or in the densities of the PCs between the L7-VGAT and control cerebellar cortices (**Table 2**). These results suggest that the loss of VGAT in the PCs did not significantly interfere with the structure of the cerebellum.

**Table 2:** Anatomical comparison between control and L7-VGAT cerebella at postnatal 40 weeks.

	Control	L7-VGAT
Molecular layer thickness (μm)	168.9 ± 5.6	174.6 ± 5.6
Granule cell layer thickness (μm)	109.7 ± 3.7	117.7 ± 1.3
PC soma density (cells/mm)	19.2 ± 1.0	19.4 ± 1.1

*All values represent mean ± SEM (*n* = 4). Student’s *t-*test was performed with genotype.*

**FIGURE 6:**
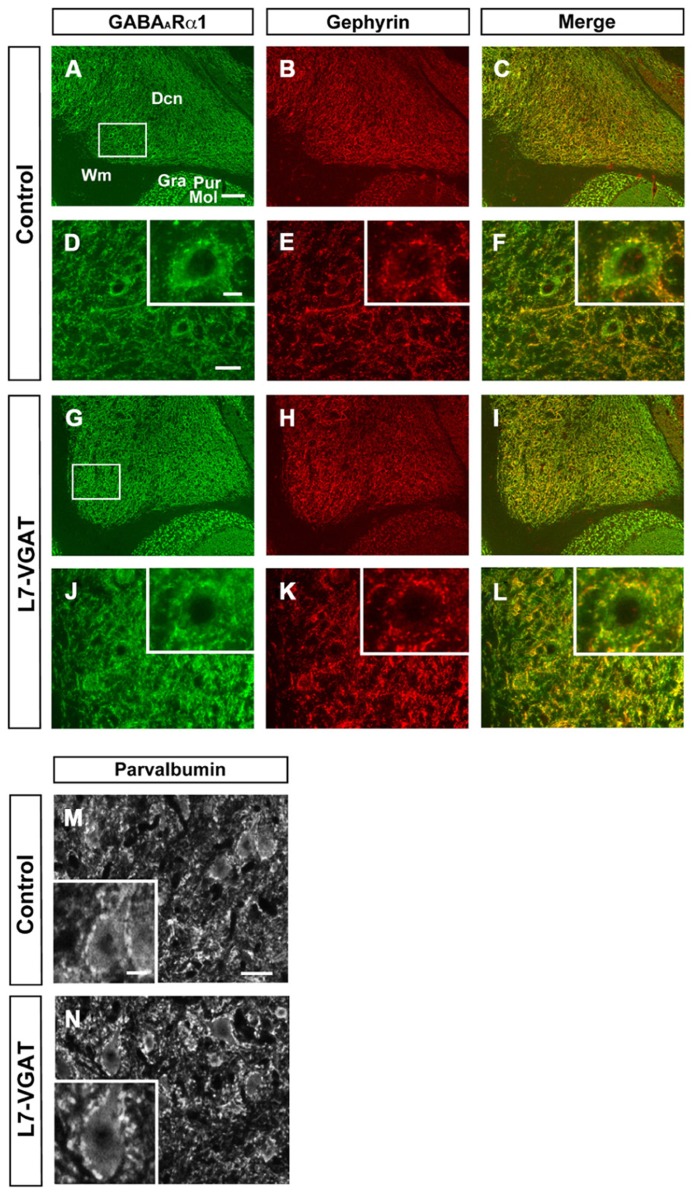
**Unaltered expression patterns of GABA_**A**_Rα1, gephyrin, and parvalbumin expression in the L7-VGAT DCN.** Immunohistochemical staining for GABA_A_Rα1 **(A,D,G,J**; green), gephyrin (**B,E,H,K**; red), and parvalbumin **(M,N)** was performed on coronal sections of the cerebella of 40-week-old mice. The DCN of control **(A–F,M)** and L7-VGAT **(G–L,N)** mice are shown. Areas surrounded by white rectangles in **(A)** and **(G)** are shown in **(D)** and **(J)**, respectively. The insets in **(D–F,J–L,M,N)** illustrate typical somata for each area. **(E,F)** and **(K,L)** shows the areas that are identical to those in **(D)** and **(J)**, respectively. Merged images of **(A,D,G,J)** and **(B,E,H,K)** are shown in **(C,F,I,L)**. Scale bars represent 100 μm **(A)**, 20 μm **(D,M)**, and 10 μm (inset of **D,M**). Dcn, deep cerebellar nucleus; Gra, granule cell layer; Pur, Purkinje cell layer; Mol, molecular layer; and Wm, white matter.

Next, we examined the immunohistochemical profiles of pre- and postsynaptic proteins at the PC-DCN synapse of the L7-VGAT cerebellum as an index of synapse formation (**Figures 3** and **6**). CB is a presynaptic protein at the PC-DCN synapse. CB immunoreactivity, including punctate signals around the somata, was largely unaltered in the L7-VGAT DCNs (**Figures 3A,D,G,J**). Moreover, PV, which is expressed in some neurons in the DCNs, was also localized to the PC terminals. The PV immunoreactive profiles were also similar between the control and L7-VGAT DCNs (**Figures 6M,N**). As representatives of postsynaptic proteins, we examined the immunoreactivities for the GABA-A receptor α1 subunit (GABA_A_Rα1, which is the most abundant subunit in adult brain including the cerebellum) and gephyrin, which is a scaffolding protein for the GABA_A_R and glycine receptor (Essrich et al., 1998; Ogris et al., 2006). These two proteins colocalized at the postsynaptic site including the somatic membrane in the control DCN (**Figures 6A–F**). Additionally, the expression patterns of GABA_A_Rα1 and gephyrin in the L7-VGAT DCNs were similar to those in the control DCNs (**Figures 6G–L**). We also performed Western blot analyses for CB, PV, GABA_A_Rα1, and gephyrin proteins in the crude fractions of the DCN to compare the expression levels of these proteins between the L7-VGAT and control mice (**Figure 4**; **Table 1**). The amounts of these four proteins were not significantly different between the genotypes. Collectively, the distribution patterns and expression levels of the representative pre- and postsynaptic proteins were not different between the L7-VGAT and control mice.

### MOTOR DISCOORDINATION IN THE L7-VGAT MICE

**FIGURE 7:**
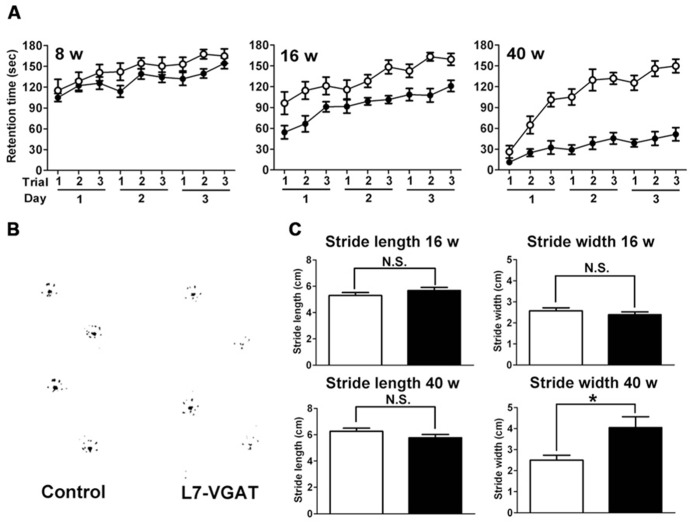
**Age-dependent motor dysfunction in L7-VGAT mice.** **(A)** Accelerating rotarod test for control (open circles) and L7-VGAT (filled circles) mice at postnatal weeks 8, 16, and 40. Latencies to fall from the rotating cylinder are plotted against each trial. The male mice were tested with three consecutive trials per day for three consecutive days. The numbers of animals were as follows: control, *n* = 14, L7-VGAT, *n* = 7 at P8W; control, *n* = 11, L7-VGAT, *n* = 9 at P16W; control, *n* = 10, L7-VGAT, *n* = 9 at P40W. Each value represents the mean± SEM. **(B)** Typical hind paw footprints for the control and L7-VGAT mice at P40W. **(C)** Hind paw stride lengths and widths at postnatal weeks 16 and 40 for control and L7-VGAT mice. Each value represents the mean± SEM. The numbers of animals were *n* = 5 in the control group (open bar) and *n* = 4 in the L7-VGAT (filled bar) group. The asterisk and N.S. represent *p* < 0.05 and *p* > 0.05, respectively (Student’s *t*-test).

Because the cerebellum is important for motor coordination, we performed accelerating rotarod tests at P8W, P16W, and P40W (**Figure 7A**). The control mice improved their performances with trial repetition, and even the older mice (P40W) demonstrated an improvement after repeated trials. At P8W, no significant differences were observed in the retention time between the L7-VGAT and control mice (*p* > 0.05), which indicates that the 8-week-old L7-VGAT mice performed normally in the accelerating rotarod test. At P16W, there was a significant difference in the retention time between the L7-VGAT and control mice (*p* < 0.001). However, there was no significant difference in the strain X trial interaction (*p* > 0.05). At P40W, there was a significant difference in the retention time between the L7-VGAT and control mice (*p* < 0.0001). Moreover, there was also a significant strain X trial interaction (*p* < 0.0001), which demonstrates that the L7-VGAT mice displayed severer ataxia at P40W than at P16W. However, the 40-week-old L7-VGAT mice began to exhibit moderately improved performance over trials (*p* < 0.0001).

Next, we examined the footprint test (**Figure 7B**). There were no significant differences in either stride length or width between the L7-VGAT and control mice at P16W (**Figure 7C**). In contrast, at P40W, the stride lengths were similar, but the stride width of the L7-VGAT mice was significantly wider than that of the control mice (L7-VGAT, 4.05± 0.52 cm; control, 2.50± 0.23 cm; *p* < 0.05) (**Figure 7C**). These results support the conclusion that the L7-VGAT mice walked normally at P16W, but displayed abnormal ambulation at P40W.

Collectively, the results of the rotarod and footprint tests demonstrate that the L7-VGAT mice displayed severer motor discoordination at P40W than at P16W.

## DISCUSSION

### THE L7-VGAT CEREBELLUM DOES NOT EXHIBIT APPARENT STRUCTURAL CHANGES

It has been reported that GABA is involved in neuronal development including the proliferation, migration, and differentiation of immature neurons (Owens and Kriegstein, 2002; Represa and Ben-Ari, 2005). However, no overt changes, including changes in lamination and foliation, were observed in the L7-VGAT cerebellum (**Figure 5**; **Table 2**). Moreover, clear alterations in the expression profiles of synaptic proteins, including CB, which is a marker protein for PCs in the cerebellum, were not observed (**Figures 3, 4**, and **6**; **Table 1**), which suggests that there were no drastic disturbances in the PC-DCN synapse including in the axon terminals of the PCs. It has been reported that there are few structural abnormalities in mice with KOs of GABAergic system-related molecules including GAD and VGAT (Ji et al., 1999; Wojcik et al., 2006). The conventional glycine receptor α2 subunit (GlyRα2) KO mice also showed no gross morphological abnormalities in the CNS including the retina (Young-Pearse et al., 2006). The acute knockdown of the *GlyRα2* expression using RNAi electroporation into the retina, however, resulted in a decrease of the rod photoreceptor cell number (Young and Cepko, 2004). In conventional KO mice in which the target gene is inactivated in a fertilized egg, a compensatory mechanism(s) may ameliorate deficits due to the absence of the molecule. Although the L7-VGAT mouse is a conditional KO one, *VGAT* expression had already disappeared in approximately 40% of the PCs at 2 weeks postnatal in the L7-VGAT cerebellum, and the progress of the KO was very slow after P2W (**Figure 2**). Therefore, it is possible that developmental compensation occurred in the L7-VGAT cerebellum. On the other hand, there are reports that suggest that neurotransmitters that exert developmental effects are released in a non-vesicular fashion (Taylor and Gordon-Weeks, 1991; Flint et al., 1998; Owens and Kriegstein, 2002). Thus, the effect of VGAT deficit on neural development may be small. It has also been reported that mutation of the *GAD67* gene causes defects in axon branching in the visual cortex during the third postnatal week, but not in the fourth postnatal week (Chattopadhyaya et al., 2007). Therefore, we cannot exclude the possibility that a transient disturbance occurred during the period before P40W in the L7-VGAT cerebellum. Studies with GABA_A_Rα1 KO mice demonstrated that substantial inhibitory synapses such as the localization of neuroligin 2 were maintained on PC dendrites of KO ones, but that the density of inhibitory synapses were significantly reduced, suggesting that synapse number is regulated by an activity-dependent manner during the period of synaptogenesis (Patrizi et al., 2008). Because GABAergic postsynaptic currents were lost in the PCs of GABA_A_Rα1 KO mice (Patrizi et al., 2008), we cannot exclude the possibility that number of synapses from the PCs was reduced in the L7-VGAT DCN, in which GABAergic outputs from PCs should be lost. Alternatively, it is possible that some disturbances would be detected at the electron microscopic level.

### ATAXIA OBSERVED IN CEREBELLAR PC-SPECIFIC VGAT-DEFICIENT MICE

L7-VGAT mice exhibited reduced retention times and widened gaits in the accelerating rotarod and footprint tests, respectively (**Figure 7**). Moreover, these impairments became more obvious as the deficit in VGAT progressed, which indicates that VGAT expression in cerebellar PCs plays an important role in the motor coordination of animals.

The cerebellum plays an important role in motor function, and PCs are indispensable for cerebellar function. The importance of PCs has been demonstrated by analyses of spontaneous mouse mutants in which cerebellar PCs are degenerated (Lalonde and Strazielle, 2007). However, the GABAergic axons of the PC that form synapses on DCN neurons disappear as a result of PC degeneration in these spontaneous mouse mutants (Bäurle et al., 1997, 1998). GABAergic actions consist of so-called phasic inhibition by vesicularly released GABA and tonic inhibition via non-vesicularly released GABA (Semyanov et al., 2004; Farrant and Nusser, 2005). Moreover, several neuropeptides, such as cholecystokinin, are expressed in PCs (Akiyama et al., 2008). Every signaling system of the PC terminals is abolished in the spontaneously PC-degenerating mice. Moreover, other types of neurons also degenerate because of the secondary effects of PC degeneration in the spontaneously PC-degenerating mouse cerebellum (Lalonde and Strazielle, 2007). Therefore, the phenotypes, including ataxia, that are observed in these spontaneously PC-degenerating mice are likely the result of many factors, which make it difficult to evaluate the significance of vesicular GABAergic neurotransmission in PCs. In contrast, neither obvious anatomical abnormalities across the entire cerebellum nor clear disturbances of the PC axon terminals on the DCNs were observed in the L7-VGAT mice (**Figures 3**–**5**; **Tables 1** and **2**). However, the L7-VGAT mice displayed ataxia, which suggests that vesicularly released GABA from the cerebellar PC terminal greatly contributed to PC function.

In the L7-VGAT mice, widened gaits were not detected at P16W when 5% of the *VGAT* mRNA-positive PCs remained, but widened gait was observed at P40W when all of the cells had disappeared (**Figures 2** and **7**). Immunoreactive signals for both CB and VGAT in the L7-VGAT DCNs at P16W were hardly detectable as they were at P40W (data not shown). These results suggest that the VGAT loss in the L7-VGAT PCs occurred at both the mRNA and protein levels during the same period. This assertion further indicates that gait disturbance can be prevented by the presence of only 5% of the normal population of vesicular GABA-releasing PCs. This inference is not compatible with the report that ataxia appears in the spontaneously PC-degenerating mutants before the number of surviving PCs is reduced by half (Landis, 1973; Mullen et al., 1976); thus, the ataxia observed in the L7-VGAT mice is less severe than that of the spontaneously PC-degenerating mutants. These findings also suggest that the compensatory effects for the dysfunction of PCs are strongly exerted in the L7-VGAT CNS (see also the previous section).

### UNALTERED-PROTEIN EXPRESSION PATTERNS AND LEVELS OF GEPHYRIN AND PARVALBUMIN

The proteins we examined as marker molecules that are present at the PC-DCN synapse include gephyrin and PV. Although the expression profiles of these proteins were not overtly altered in the L7-VGAT DCN (**Figures 3, 4**, and **6**; **Table 1**), those in the DCN and VN of the spontaneously PC-degenerating mutants were altered. The number of puncta that were immunoreactive for gephyrin was reduced by approximately half (Garin et al., 2002), and PV-positive somata appeared (Bäurle et al., 1997, 1998).

Gephyrin clustering at the postsynaptic sites is induced by α-neurexin in the presynaptic site (Kang et al., 2008). When α-neurexin is bound with neuroligin 2, gephyrin associates with neuroligin 2 and collybistin to form a complex and is involved in the clustering of inhibitory neurotransmitter receptors (Poulopoulos et al., 2009). The PC axons are retracted from synaptic sites in PC-degenerating mutants, and α-neurexin in the PC axon terminals are far from the synaptic sites. Collectively, it is likely that the retraction of PC axons, rather than the loss of output from PCs, inhibits the formation of the neurexin–neuroligin complex and affects gephyrin clustering at postsynaptic sites in PC-degenerating mutants.

PV is a Ca^2^^+^-binding protein that buffers intracellular Ca^2^^+^. It is thought that the concentration of intracellular PV reflects the activity level of the neuron (Kamphuis et al., 1989; Bäurle et al., 1998; Chaudhury et al., 2008). Although many DCN neurons express PV, PV-immunoreactivity is detected only when axonal transport is pharmacologically inhibited (Celio, 1990; Bäurle et al., 1998). Moreover, most PV-positive cells are inhibitory neurons in the DCN of *PCD* mutant mice (Bäurle et al., 1997). Moreover, BBäurle et al. (1998) asserted that the activity of inhibitory neurons in the DCN should increase to compensate for the reduced inhibitory input from the PCs to the DCN, which was accompanied by a change in PV expression in the spontaneous mutants. However, neither the appearance of PV-positive somata nor significant elevations of PV protein levels were observed in the L7-VGAT DCN (**Figures 4 and 6**; **Table 1**). In the L7-VGAT DCN, the loss of GABAergic inhibition from PCs is expected. Nevertheless, the elevated expression of PV did not occur, which implies that the reorganization of the somatic motor pathway that occurs in the L7-VGAT CNS differs from that in the spontaneous mutants.

### L7-VGAT MICE AS A MODEL OF THE PC-DYSFUNCTIONAL MUTANT

The L7-VGAT mice generated in this study became completely PC-specific VGAT knockout mice and exhibited ataxia. It was expected that only vesicular release of GABA from the PCs would be disrupted in the L7-VGAT cerebellum and that PC degeneration and other drastic changes in cerebellar structure would not occur. The majority of mouse models of ataxia display histological phenomena, such as losses of PCs, which explain their cerebellar dysfunctions. Only a few mouse models exist that display ataxic behavior combined with normal cerebellar morphology (Hendriks et al., 2009). Additionally, the cerebellum has been reported to be critical also for cognition and emotion (Schmahmann, 2010). Therefore, the L7-VGAT mice will greatly contribute to studies of cerebellar functions from the cellular level (e.g., neuronal connectivity) to the whole-body level (e.g., motor coordination, vestibular compensation, cognition, and emotion).

The VGAT deficit was complete in the PCs at P40W (**Figures 2** and **3**). Because the L7-Cre gene in the L7-VGAT mice was heterozygous, the VGAT-flox gene would be expected to be deleted much earlier in the animals that are homozygous for the L7-Cre gene. The use of these two lines of L7-VGAT mice may be useful in the study of the differences in the effect of VGAT deficits that occur at different ages.

### CONCLUSION

Studies using L7-VGAT mice demonstrated that VGAT in cerebellar PCs was not essential for the maintenance of overall cerebellar structure or the expression profiles of synaptic proteins including calbindin, parvalbumin, GABA_A_Rα1, and gephyrin, but that it was crucial for the motor coordination in animals. The L7-VGAT mice will be a useful model to provide a clue to the better understanding of PC function in diverse cerebellum-related behaviors.

## AUTHOR CONTRIBUTIONS

Mikiko Kayakabe, Toshikazu Kakizaki, Ryosuke Kaneko, Yoichi Nakazato, Koji Shibasaki, Yasuki Ishizaki, Nobuhiko Furuya, and Yuchio Yanagawa: Conceived and designed the experiments; Mikiko Kayakabe, Toshikazu Kakizaki, Ryosuke Kaneko, Atsushi Sasaki, Koji Shibasaki, and Yuchio Yanagawa: Performed the experiments; Mikiko Kayakabe, Toshikazu Kakizaki, Ryosuke Kaneko, Atsushi Sasaki, Koji Shibasaki, and Yuchio Yanagawa: Analyzed the data; Ryosuke Kaneko, Atsushi Sasaki, Yoichi Nakazato, Hiromitsu Saito, and Noboru Suzuki: Contributed new reagents/analytical tools; Mikiko Kayakabe, Toshikazu Kakizaki, Ryosuke Kaneko, and Yuchio Yanagawa:Wrote the paper. All authors read and approved the final manuscript.

## Conflict of Interest Statement

The authors declare that the research was conducted in the absence of any commercial or financial relationships that could be construed as a potential conflict of interest.

## Abbreviations

BSA: bovine serum albumin;
CB: calbindin;
CNS: central nervous system;
DCN: deep cerebellar nuclei;
DIG: digoxigenin;
ECL: enhanced chemiluminescence;
EDTA: ethylenediaminetetraacetic acid;
GABA: γ-aminobutyric acid;
GABA_A_Rα1: GABA-A receptor α1 subunit;
GAD: glutamate decarboxylase;
GAT3: GABA transporter 3;
GlyRα2: glycine receptor α2 subunit;
IPSC: inhibitory postsynaptic current;
ISH: *in situ* hybridization;
KO: knockout;
PB: phosphate buffer;
PBSTx: Triton X-100 in PBS;
PC: Purkinje cell;
PFA: paraformaldehyde;
PMSF: phenylmethylsulfonyl fluoride;
PV: parvalbumin;
P40W: postnatal 40 weeks of age;
SDS–PAGE: sodium dodecyl sulfate–polyacrylamide gel electrophoresis;
VGAT: vesicular GABA transporter;
VN: vestibular nuclei

## References

[B1] Akiyama K. Nakanishi S. Nakamura N. H. Naito T. (2008). Gene expression profiling of neuropeptides in mouse cerebellum, hippocampus, and retina. *Nutrition* 24 918–923 10.1016/j.nut.2008.06.018 18662864

[B2] Bäurle J. Helmchen C. Grusser-Cornehls U. (1997). Diverse effects of Purkinje cell loss on deep cerebellar and vestibular nuclei neurons in Purkinje cell degeneration mutant mice: a possible compensatory mechanism. *J. Comp. Neurol.* 384 580–596 10.1002/(SICI)1096-9861(19970811)384:4<580::AID-CNE7>3.0.CO;2-Z 9259491

[B3] Bäurle J. Hoshi M. Grusser-Cornehls U. (1998). Dependence of parvalbumin expression on Purkinje cell input in the deep cerebellar nuclei. *J. Comp. Neurol.* 392 499–514 10.1002/(SICI)1096-9861(19980323)392:4<499::AID-CNE7>3.0.CO;2-W 9514513

[B4] Buddhala C. Hsu C. C. Wu J. Y. (2009). A novel mechanism for GABA synthesis and packaging into synaptic vesicles. *Neurochem. Int.* 55 9–12 10.1016/j.neuint.2009.01.020 19428801

[B5] Celio M. R. (1990). Calbindin D-28k and parvalbumin in the rat nervous system. *Neuroscience* 35 375–475 10.1016/0306-4522(90)90091-H 2199841

[B6] Chattopadhyaya B. Di Cristo G. Wu C. Z. Knott G. Kuhlman S. Fu Y. (2007). GAD67-mediated GABA synthesis and signaling regulate inhibitory synaptic innervation in the visual cortex. *Neuron* 54 889–903 10.1016/j.neuron.2007.05.015 17582330 PMC2077924

[B7] Chaudhury S. Nag T. C. Wadhwa S. (2008). Calbindin D-28K and parvalbumin expression in embryonic chick hippocampus is enhanced by prenatal auditory stimulation. *Brain Res.* 1191 96–106 10.1016/j.brainres.2007.11.021 18096144

[B8] Ebihara S. Obata K. Yanagawa Y. (2003). Mouse vesicular GABA transporter gene: genomic organization, transcriptional regulation, and chromosomal localization. *Mol. Brain Res.* 110 126–139 10.1016/S0169-328X(02)00648-4 12573541

[B9] Essrich C. Lorez M. Benson J. A. Fritschy J. M Lüscher B. (1998). Postsynaptic clustering of major GABAA receptor subtypes requires the gamma 2 subunit and gephyrin. *Nat. Neurosci.* 7 563–571 10196563 10.1038/2798

[B10] Farrant M. Nusser Z. (2005). Variations on an inhibitory theme: phasic and tonic activation of GABA(A) receptors. *Nat. Rev. Neurosci.* 6 215–229 10.1038/nrn1625 15738957

[B11] Flint A. C. Liu X. Kriegstein A. R. (1998). Nonsynaptic glycine receptor activation during early neocortical development. *Neuron* 1 43–53 10.1016/S0896-6273(00)80433-X 9459441

[B12] Garin N. Hornung J. P. Escher G. (2002). Distribution of postsynaptic GABA(A) receptor aggregates in the deep cerebellar nuclei of normal and mutant mice. *J. Comp. Neurol.* 447 210–217 10.1002/cne.10226 11984816

[B13] Hendriks W. J. Dilaver G. Noordman Y. E. Kremer B. Fransen J. A. (2009). PTPRR protein tyrosine phosphatase isoforms and locomotion of vesicles and mice. *Cerebellum* 8 80–88 10.1007/s12311-008-0088-y 19137382 PMC2694922

[B14] Itouji A. Sakai N. Tanaka C. Saito N. (1996). Neuronal and glial localization of two GABA transporters (GAT1 and GAT3) in the rat cerebellum. *Brain Res. Mol. Brain Res.* 37 309–316 10.1016/0169-328X(95)00342-P 8738166

[B15] Ji F. Kanbara N. Obata K. (1999). GABA and histogenesis in fetal and neonatal mouse brain lacking both the isoforms of glutamic acid decarboxylase. *Neurosci. Res.* 33 187–194 10.1016/S0168-0102(99)00011-5 10211762

[B16] Kamphuis W. Huisman E. Wadman W. J. Heizmann C. W Lopes da Silva F. H. (1989). Kindling induced changes in parvalbumin immunoreactivity in rat hippocampus and its relation to long-term decrease in GABA-immunoreactivity. *Brain Res.* 479 23–34 10.1016/0006-8993(89)91331-0 2924151

[B17] Kang Y. Zhang X. Dobie F. Wu H. Craig A. M. (2008). Induction of GABAergic postsynaptic differentiation by alpha-neurexins. *J. Biol. Chem.* 283 2323–2334 10.1074/jbc.M703957200 18006501 PMC2811689

[B18] Lalonde R. Strazielle C. (2007). Spontaneous and induced mouse mutations with cerebellar dysfunctions: behavior and neurochemistry. *Brain Res.* 1140 51–74 10.1016/j.brainres.2006.01.031 16499884

[B19] Landis S. C. (1973). Ultrastructural changes in the mitochondria of cerebellar Purkinje cells of nervous mutant mice. *J. Cell. Biol.* 57 782–797 10.1083/jcb.57.3.782 4698906 PMC2109013

[B20] Lisberger S. G. Thach W. T. (2013). “The cerebellum,” in *Principles of Neural Science* 5th Edn. eds Kandel E. R. Schwartz J. H. Jessell T. M. Siegelbaum S. A. Hudspeth A. J. (New York: McGraw-Hill) 960–981

[B21] McIntire S. L. Reimer R. J. Schuske K. Edwards R. H. Jorgensen E. M. (1997). Identification and characterization of the vesicular GABA transporter. *Nature* 389 870–876 10.1038/39908 9349821

[B22] Mullen R. J. Eicher E. M. Sidman R. L. (1976). Purkinje cell degeneration, a new neurological mutation in the mouse. *Proc. Natl. Acad. Sci. U.S.A.* 73 208–212 10.1073/pnas.73.1.208 1061118 PMC335870

[B23] Ogris W. Lehner R. Fuchs K. Furtmüller B. Höger H. Homanics G. E. (2006). Investigation of the abundance and subunit composition of GABAA receptor subtypes in the cerebellum of alpha1-subunit-deficient mice. *J. Neurochem.* 96 136–147 10.1111/j.1471-4159.2005.03509.x 16277610

[B24] Owens D. F. Kriegstein A. R. (2002). Is there more to GABA than synaptic inhibition? *Nat. Rev. Neurosci.* 3 715–727 10.1038/nrn919 12209120

[B25] Patrizi A. Scelfo B. Viltono L. Briatore F. Fukaya M. Watanabe M. (2008). Synapse formation and clustering of neuroligin-2 in the absence of GABAA receptors. *Proc. Natl. Acad. Sci. U.S.A.* 105 13151–13156 10.1073/pnas.0802390105 18723687 PMC2529038

[B26] Poulopoulos A. Aramuni G. Meyer G. Soykan T. Hoon M. Papadopoulos T. (2009). Neuroligin 2 drives postsynaptic assembly at perisomatic inhibitory synapses through gephyrin and collybistin. *Neuron* 63 628–642 10.1016/j.neuron.2009.08.023 19755106

[B27] Represa A. Ben-Ari Y. (2005). Trophic actions of GABA on neuronal development. *Trends Neurosci.* 28 278–283 10.1016/j.tins.2005.03.010 15927682

[B28] Sagn𠃩 C. El Mestikawy S. Isambert M. F. Hamon M. Henry J. P. Giros B. (1997). Cloning of a functional vesicular GABA and glycine transporter by screening of genome databases. *FEBS Lett.* 417 177–183 10.1016/S0014-5793(97)01279-9 9395291

[B29] Saito H. Tsumura H. Otake S. Nishida A. Furukawa T. Suzuki N. (2005). L7/Pcp-2-specific expression of Cre recombinase using knock-in approach. *Biochem. Biophys. Res. Commun.* 331 1216–1221 10.1016/j.bbrc.2005.04.043 15883005

[B30] Saito K. Kakizaki T. Hayashi R. Nishimaru H. Furukawa T. Nakazato Y. (2010). The physiological roles of vesicular GABA transporter during embryonic development: a study using knockout mice. *Mol. Brain* 3 40 10.1186/1756-6606-3-40 PMC3023674 21190592

[B31] Semyanov A. Walker M. C. Kullmann D. M. Silver R. A. (2004). Tonically active GABA A receptors: modulating gain and maintaining the tone. *Trends Neurosci.* 27 262–269 10.1016/j.tins.2004.03.005 15111008

[B32] Schmahmann J. D. (2010). The role of the cerebellum in cognition and emotion: personal reflections since 1982 on the dysmetria of thought hypothesis, and its historical evolution from theory to therapy. *Neuropsychol. Rev.* 20 236–260 10.1007/s11065-010-9142-x 20821056

[B33] Strata P. Scelfo B. Sacchetti B. (2011). Involvement of cerebellum in emotional behavior. *Physiol. Res.* 60(Suppl. 1) S39–S48 10.33549/physiolres.932169 21777033

[B34] Takayama C. Inoue Y. (2004a). Extrasynaptic localization of GABA in the developing mouse cerebellum. *Neurosci. Res.* 50 447–458 10.1016/j.neures.2004.08.012 15567482

[B35] Takayama C. Inoue Y. (2004b). Morphological development and maturation of the GABAergic synapses in the mouse cerebellar granular layer. *Brain Res. Dev. Brain Res.* 150 177–190 10.1016/j.devbrainres.2004.03.011 15158081

[B36] Takeuchi T. Miyazaki T. Watanabe M. Mori H. Sakimura K. Mishina M. (2005). Control of synaptic connection by glutamate receptor delta2 in the adult cerebellum. *J. Neurosci.* 25 2146–2156 10.1523/JNEUROSCI.4740-04.2005 15728855 PMC6726062

[B37] Taylor J. Gordon-Weeks P. R. (1991). Calcium-independent gamma-aminobutyric acid release from growth cones: role of gamma-aminobutyric acid transport. *J. Neurochem.* 56 273–280 10.1111/j.1471-4159.1991.tb02592.x 1987321

[B38] Tong Q. Ye C. P. Jones J. E. Elmquist J. K. Lowell B. B. (2008). Synaptic release of GABA by AgRP neurons is required for normal regulation of energy balance. *Nat. Neurosci.* 11 998–1000 10.1038/nn.2167 19160495 PMC2662585

[B39] Watakabe A. Ichinohe N. Ohsawa S. Hashikawa T. Komatsu Y. Rockland K. S. (2007). Comparative analysis of layer-specific genes in mammalian neocortex. *Cereb. Cortex* 17 1918–1933 10.1093/cercor/bhl102 17065549

[B40] Watakabe A. Komatsu Y. Ohsawa S. Yamamori T. (2010). Fluorescent in situ hybridization technique for cell type identification and characterization in the central nervous system. *Methods* 52 367–374 10.1016/j.ymeth.2010.07.003 20637287

[B41] Wojcik S. M. Katsurabayashi S. Guillemin I. Friauf E. Rosenmund C. Brose N. (2006). A shared vesicular carrier allows synaptic corelease of GABA and glycine. *Neuron* 50 575–587 10.1016/j.neuron.2006.04.016 16701208

[B42] Young-Pearse T. L. Ivic L. Kriegstein A. R. Cepko C. L. (2006). Characterization of mice with targeted deletion of glycine receptor alpha 2. *Mol. Cell. Biol.* 26 5728–5734 10.1128/MCB.00237-06 16847326 PMC1592777

[B43] Young T. L. Cepko C. L. (2004). A role for ligand-gated ion channels in rod photoreceptor development. *Neuron* 41 867–879 10.1016/S0896-6273(04)00141-2 15046720

